# Sleep and COVID-19: considerations about immunity, pathophysiology, and treatment

**DOI:** 10.5935/1984-0063.20200062

**Published:** 2020

**Authors:** Marco Túlio De Mello, Andressa Silva, Renato de Carvalho Guerreiro, Flavia Rodrigues da-Silva, Andrea Maculano Esteves, Dalva Poyares, Ronaldo Piovezan, Erika Treptow, Marcelo Starling, Daniela Santoro Rosa, Gabriel Natan Pires, Monica Levy Andersen, Sergio Tuﬁk

**Affiliations:** 1 Universidade Federal de Minas Gerais, Departamento de Esportes - Belo Horizonte - Minas Gerais - Brazil.; 2 Universidade Estadual de Campinas, Faculdade de Ciências Aplicadas - Limeira - São Paulo - Brazil.; 3 Universidade Federal de São Paulo, Departamento de Psicobiologia - São Paulo - São Paulo - Brazil.; 4 Federal University of São Paulo, Departament of Microbiology, Imunology and Parasitology - São Paulo - São Paulo - Brazil.; 5 Santa Casa de São Paulo School of Medical Sciences, Department of Physiological Sciences - São Paulo - São Paulo - Brazil.

**Keywords:** Sleep, Sleep Wake Disorders, SARS Virus, Coronavirus Infections, Immune System, Therapeutics

## Abstract

The fear and uncertainty caused by the coronavirus disease 2019 (COVID-19) pandemic, threats to survival are one of the main problems of everyday life; however, mental health care must also be considered a priority. During social isolation also called self-quarantine, the restricted mobility and social contact, concern about ﬁnancial resources and availability of supplies, fear of infection, questions about the duration of self-quarantine, cause anxiety, depression, stress, insomnia and reduced the quality and quantity of sleep, that may present a greater risk to the health of the general population. Sleep disorders are increasingly becoming a major health issue in modern society, and are inﬂuenced by retinal stimulation by electronic devices, as well extended and/or night shift-work, which may aggravate the systemic and lung inﬂammation during viral infections. Sleep disorders can induce pro-inﬂammatory states and be harmful during the COVID-19 pandemic. The possible interactions between many drugs used to treat COVID-19, and those used to treat sleep disorders are unknown, mostly due to the lack of a standard protocol to treat these patients. Insufficient sleep or irregular sleep-wake cycles may impair health, immune system, induce pro-inﬂammation state, and may lead to increased vulnerability to viral infections, involving inﬂammatory and oxidative/antioxidant imbalance. In this sense, obstructive sleep apnea has been associated with recognized COVID-19 risk comorbidities and considered a risk factor for COVID-19. During the COVID-19 pandemic, health care cannot stop, and telemedicine has presented itself as an alternative method of delivering services. When a face-to-face visit is mandatory, or in locations with minimal community transmission where sleep centers have resumed activities, it is important that the sleep center facilities are properly prepared to receive the patients during the COVID-19 pandemic, and follow all relevant safety rules. In this work we gathered a group of researchers, specialists in aspects related to chronobiology, sleep, sleep disorders, and the immune system. Thus, we conducted a narrative review in order to address the relationship between COVID-19 and sleep, as well as its immunological aspects and strategies that may be applied in order to mitigate the harmful effects on health that affects everyone during the pandemic.

## INTRODUCTION

Sleep is a physiological and behavioral state essential to life, considered to play an essential role in the homeostasis, immune system, maintaining performance, muscle restoration, energy metabolism, cognitive function, and neural plasticity^^1^^. It has been recommended that adult sleep duration should be between seven and nine hours per night^^2^^. However, in recent decades there has been a reduction of approximately two to three hours in the duration of sleep in several populations around the world, as well as an increase of the number of people who work at night whose biological rhythm has become inverted^^3^,^4^^.

Sleep deprivation (SD) leads to decreased physical and cognitive performance, reduced alertness, and has a negative impact on health including increased risk of stroke, obesity, diabetes, cancer, osteoporosis, and cardiovascular disease^^5^^. The causes of SD are wide-ranging and not just related with poor sleep habits and shift-work, i.e., sleep disorders, age, illness, environmental, etc. Non-standard working hours may also cause physiological alterations, and even result in circadian rhythm disturbances, as is common among shift-workers. In addition, medical problems (such as osteoarthritis) and sleep disorders (e.g., insomnia, restless legs syndrome (RLS), obstructive sleep apnea (OSA)) are observed^^5^,^6^^.

In the current scenario caused by the pandemic of coronavirus disease 2019 (COVID-19), sleep is an important component for the maintenance of a functional immune system and the health of the population. Since the beginning of the pandemic, population has been subjected to a substantial period of social isolation with movement restrictions also called self-quarantine^^7^^. Self-quarantine has been adopted as a measure to reduce the spread of the disease, and there have been significant changes in people’s lifestyles^^8^^, with some studies reporting a reduction in sleep quality. This has been associated with stress, increased exposure to artificial light (including blue LED - light-emitting diode-exposure at night), and reduced exposure to sunlight during the day^^8^,^9^^.

Other studies have reported an increase in body weight, related to increased food intake in response to mental stress and spend more time at home, increased opportunity to feed, increased visual and olfactory stimulation to eat^^7^^. Short sleep duration per night, as well as the less amount of physical activity undertaken, are considered to be predictors of weight gain^^7^^. There is growing evidence of a relationship between reduced sleep quality, increased anxiety and mental stress, increased weight, and reduced physical activity due to COVID-19 and social isolation. This review aims to discuss issues related to sleep and COVID-19, especially for groups that already had problems related to sleep and were receiving pharmacological or non-pharmacological treatments. Thus, this review was organized in a way that will present an overview of COVID-19 in today’s society as well as the mechanisms of a new coronavirus (SARS-CoV-2). In the second part, we will discuss some sleep disorders that can be aggravated during the pandemic as well as possibilities for diagnoses and treatments at the present time. In a third part, it will be discussed how sleep deprivation and circadian misalignment can affect the immune system and how this interaction can be a risk during the pandemic. It will also be presented how the exercise can be an ally or a villain depending on how it is performed. Finally, we will address a perspective for the future when facing COVID-19.

## COVID-19 pandemic: unprecedent episode about today´s society

Coronaviruses (CoVs) are a group of single-stranded RNA viruses that, which are able to infect humans. Six human CoVs have been identified and in 2019, SARS-CoV-2 emerged in Wuhan, China, and rapidly spread worldwide causing a pandemic^^10^^. Similarly, to SARS and MERS, SARS-CoV-2 affects the respiratory tract and can cause pneumonia and consequently, respiratory failure^^11^^.

Older individuals or people with comorbidities including obesity, diabetes, hypertension, heart disease, chronic kidney disease, cancer, and chronic obstructive lung disease are more susceptible to COVID-19^^12^-^16^^.

Severe cases of COVID-19 are often related with higher levels of D-dimer, lactate dehydrogenase, C-reactive protein (CRP), ferritin, interleukin (IL)-6, IL-10, IL-2R (soluble IL-2 receptor), and tumor necrosis factor alpha (TNF-a), which are also accompanied by an increased white blood cell counts (higher neutrophil counts) but lower absolute numbers of T helper (CD4+) and T cytotoxic (CD8+) lymphocytes, and decreased interferon gamma (IFNγ) expression by CD4+ T cells^^17^,^18^^. Furthermore, COVID-19 has been linked to immune cell infiltration into the lungs (mainly neutrophils and monocytes) and high levels of chemokines such as interferon-inducible protein 10 (CXCL10), monocyte chemotactic protein-1 (CCL2), and macrophage inflammatory protein 1 alpha (CCL3)^^15^^. Hence, the overproduction of soluble markers results in a cytokine storm that seems to be proportional to disease severity^^18^^.

Due to the highly infectious nature of the disease and its fast rate of transmission, societies mobilized to limit travel and adopt social isolation policies. The pandemic sparked a wave of fear and uncertainty, not only related to the infection itself, but also to its social consequences, such as unemployment and financial hardship. Among the main factors associated with the decline in mental health during self-quarantine and social isolation are: restricted mobility and social contact, concern about financial resources and availability of supplies, fear of infection, uncertainty about the duration of self-quarantine period - all of which can all lead to increased anxiety, depression, and stress^^19^-^21^^.

These symptoms may directly affect the amount and quality of sleep in the population. Studies report that people began sleeping more and more regularly every night after countries imposed stay-at-home orders to slow the spread of the coronavirus, but may not have been of the best quality^^22^^. Inclusive, it has been demonstrated that in three European countries (Switzerland, Germany, and Austria), the COVID-19 lockdown led to improved individual sleep-wake timing and overall more sleep. At the same time, however, many people suffered from a decrease in sleep quality in this burdening and exceptional situation^^23^^. In this sense, a reduction in sleep duration associated with the stress and anxiety caused by the self-quarantine has already been reported^^8^^. Social isolation and the other psychological impacts of the disease may not simply disrupt sleep, as changes in sleep patterns can have significant consequences for a wide range of health risks in the general population. Thus, this review addresses the relationship between COVID-19 and sleep, as well as its immunological aspects and strategies that can be used in order to mitigate the harmful effects on health that affects everyone during the pandemic.

## Sleep disorders during COVID-19 pandemic

Sleep disorders is increasingly becoming a major problem in modern society due to its effects on health, with organ systems in particular being negatively affected^^24^^. Many factors, such as hyper stimulation by the media being TV, newspapers and radio, electronic devices, shift-work, and psychological disorders, such as stress and anxiety, are significant contributors to acute and chronic SD. Several studies, have linked short sleep duration^^25^,^26^^, shift-work^^27^,^28^^, acute^^29^,^30^^, and chronic^^31^,^32^^ SD with diseases^^33^^. 

Sleep maintains regular expression over a 24-hour period of the levels of the cells, such as monocytes, macrophages, and dendritic cells, which form part of the immune system. However, sleep disturbances can modify their expression, for example, levels of pro-inflammatory IL-6 normally peak at 7:00 p.m. and at 5:00 a.m., but nighttime SD can delay or reduce IL-6 secretion proportionally to the amount of sleep loss^^34^,^35^^, as well as monocytes increase and their ability to respond to microbial challenges during sleep, specifically by secretion of tumor necrosis factor-α (TNF-α), which raises the converse possibility that lack of sleep impairs this effect^^36^,^37^^. In this sense, both acute (4-night vigil) or partial (4.2-h sleep for 10 days) SD, induce an increase of CRP levels impacting systolic blood pressure and heart rate^^38^^.

Although SD alone does not significantly alter immune cell numbers^^39^^, chronic insomnia has been shown to alter the relative distribution of immune cell phenotypes, with a steep decrease in CD3+, CD4+, and CD8+ cell counts, for example^^40^^. SD can exert a strong influence on cytokine balance, which, in turn, profoundly changes the immune response profile. Importantly, partial and total SD for one night has been shown to reduce T-cell production of IL-2^^41^-^43^^, while continuous SD has been associated with a shift in cytokine balance favoring Th2 cytokine activity over Th1^^44^-^48^^, skewing the immune response toward B-cell activation. This shift toward a Th2 response can have deleterious consequences in the case of infection, or when the organism is artificially challenged with an antigen during vaccination. Regarding this point, one night of partial SD reduce immunologic response to influenza A and hepatitis A vaccination^^49^^.

Similar SD, stress and loneliness being associated with a reduced antibody response against influenza and hepatitis B vaccinations^^50^-^52^^, which need attention during the COVID-19 pandemic. Furthermore, SD in man, but not in females, reduce serum antibody levels five days after immunization^^53^^. Regarding infection, short sleep duration is capable to reduce the resistance against rhinovirus^^54^,^55^^.

Given these data regarding alterations in immune response profile, lower immunization efficacy, and higher susceptibility to infection, it is not unreasonable to assume SD may enhance the risk of individuals becoming infected by SARS-CoV-2, and lower immune defense against COVID-19 disease. Even if this proves not to be true, making such an assumption during the pandemic would be, at the very least, irresponsible. Hence, during these challenging times, as a precautionary principle one cannot afford to risk increasing an individual’s (and those they have close contact with) chance of contamination, and the strength of their immune response, by ignoring the potential role that sleep may well play in relation to COVID-19.

### Obstructive sleep apnea - OSA and its pathophysiological aspects in the context of COVID-19 pandemic

Obstructive sleep apnea (OSA) is a sleep breathing disorder characterized by intermittent partial or total obstructions of the upper airway during sleep. It is estimated that 936 million adults aged 30-69 years have OSA globally^^56^^, and the prevalence may be higher in older adults^^57^^. OSA is associated with comorbidities considered risk factors for patients with COVID-19, such as hypertension, diabetes, cardiovascular disorders, and obesity^^58^-^60^^. However, mortality and hospitalizations are not fully explained by these recognized risk factors. Given the association of OSA with comorbidities, recognized as risk factor for COVID-19, a recent study identified OSA as a risk factor for COVID-19 mortality^^61^^. It is believed that OSA had not been identified as a risk factor due to its clinical importance for this situation to be under-recognized. Thus, the importance of better efforts to recognize sleep apnea in individuals with COVID-19 infection was recommended^^61^^. Furthermore, sleep disorders, including OSA, can be related with immunological and inflammatory factors involved in the morbimortality, which deserve attention to be investigated it relationships with the infection by SARS-CoV-2. Here, we discuss the possible pathophysiological aspects of OSA potentially implicated in the clinical course of COVID-19, and current recommendations on the management of OSA during these challenging times.

Sleep disorders, as OSA can aggravate, hypothetically, the systemic and lung inflammation that can occur during viral infections, and perhaps, including COVID-19^^62^^. It because OSA leads to chronic intermittent unbalance between excessive sympathetic activation and parasympathetic activity reduction, as well as hypoxia, all of them potentially increasing IL-6, TNF-α, and IL1-β levels^^63^^. Similarly, an exacerbated inflammatory response resulting in damage to the airways is the most common manifestation of the infection by SARS-CoV-2. As a result, severe COVID-19 progresses to acute respiratory distress syndrome ^^64^^. 

The acute and massive release of pro-inflammatory cytokines in response to viral infection and/or secondary bacterial infections increases the risk of a cytokine storm. This uncontrolled inflammation can result in multi-organ failure, with patients with SARS-CoV-2 infection progressing to renal failure being more likely to die^^65^^. Importantly, the pro-inflammatory states observed in some individuals with OSA could be related to the risk of negative outcomes if infected with COVID-19. Although, to the best of our knowledge, there are no published studies with consistent number of patients and with respective follow-up directly addressing OSA as a risk factor for an adverse clinical course in those affected by COVID-19, some evidence looking at the risk factors for the aggravation of respiratory viral infections has shown that hospitalized patients with OSA were more likely to require intensive care unit admission^^66^^.

Conversely, the respiratory and cardiovascular consequences of COVID-19 can worsen, or even lead to, transient new OSA cases due to the inflammation of the upper way and the acute and persistent hypoxia caused by the infection. As mentioned above, further studies targeting the relationships between OSA and COVID-19 are warranted to provide additional evidence regarding the role of this sleep breathing disorder in the course of SARS-CoV-2 infection^^67^^.

### Diagnosis of OSA during COVID-19 pandemic

As the prevalence and risk of COVID-19 increased worldwide, many sleep centers were closed to minimize the spread of the virus. This means that the initial consultation now has to be undertaken using telemedicine. Home studies can then be used as the preferred method of diagnosis, with the equipment being delivered by mail to the patients to avoid contact with others. In order to reduce the risk of infection, disposable sensors should be use. If those are not available, the recommendation is to follow the manufactures’ instructions in regard of the cleaning process, and then wait at least 72 hours before reusing the equipment due to the persistence of SARS-CoV-2 on surfaces^^68^,^69^^.

The emphasis of the guidelines is on avoiding doctor to patient contact during the pandemic. The American Academy of Sleep Medicine has issued mitigation strategies to help sleep medicine clinicians in their professional activities during the spread of COVID-19^^70^^. Their recommendations are based on the recommendations of Centers for Disease Control and Prevention to contain and avert infection of COVID-19 in health-care settings^^71^^. European Sleep Research Society has published guidelines on how to manage sleep during COVID-19, especially pertaining to cognitive behavioral therapy for insomnia^^72^^. The British Sleep Society has made statements on sleep-related advice to patients during the COVID-19 pandemic^^73^^. The American Clinical Neurophysiology Society guidelines describe in detail about technologist safety and staffing, equipment maintenance and cleaning, managing requests for neurodiagnostic testing, and physician staffing^^74^^. All the guidelines discussed above emphasis on avoiding doctor to patient contact during any pandemic. Home sleep testing is gaining more attention during the COVID-19 pandemic, which various levels of sleep studies are the major aspects in these guidelines.

Regarding this, when a face-to-face visit is necessary, or in locations with minimal community transmission where sleep centers have resumed activities, it is important that the sleep center facilities be prepared to receive the patients. It is recommended to avoid long stays in the waiting room, provide hand soap and hand sanitizer in all areas, minimize contact with others, and remove any non-essential items from the rooms, in which the sleep test will be performed. Screening of patients prior to the visit and at arrival for potential symptoms is essential, and temperature checks for patients and workers are recommended. Sleep technologists and physicians should wear personal protective equipment, and the patients should wear a mask. 

Sleep clinicians should be prepared to adjust operations as local conditions change. Short-term closure of the sleep center may be needed in response to the surge in local community transmission. In this sense, sleep studies during the COVID-19 pandemic may be conducted in the laboratory or at home, depending on the level of the community spread and the condition of the sleep disorder^^75^^.

### The treatment of OSA during the COVID-19 pandemic

Continuous positive airway pressure (CPAP) is a modality of non-invasive ventilation and the mainstay therapy for OSA. CPAP provides a positive airflow that acts as a pneumatic splint to maintain upper airway patency during sleep. The connection of the equipment to the patient requires an interface (mask) that has an intentional leak to allow the patient to exhale CO2. However, this intentional leak means that non-invasive ventilation is an aerosol-generating therapy, and exhaled particles may remain in the air for more than one hour and increase the risk of contamination to people in the same area^^76^,^77^^. The decision to maintain or temporarily suspend CPAP should be done on a case-by-case basis, taking into consideration the severity of OSA, the presence of comorbidities, and the risks of stopping treatment^^78^,^79^^.

A general approach to the management of OSA includes the control of body weight. Prolonged home confinement, physical inactivity, unhealthy eating habits and anxiety can result in weight gain^^7^^ and potentially increase the severity of OSA^^80^^. A healthy diet with a balanced consumption of macronutrients (fats, carbohydrates, and protein) and regular low-intensity exercise are recommended. Patients should be aware that the consumption of alcoholic beverages in the evening are associated with increased respiratory obstructive events, oxygen desaturation, and sleep fragmentation^^81^^. 

Myofunctional therapy via telemedicine can be used for mild and moderate cases, as can positional therapy. Treatment with a mandibular advancement device is a possible alternative therapy during the pandemic, especially for patients that already have a device. However, mandibular advancement device treatment should be restricted to localities where the specialists involved in the patient’s care remain active (dentists, prosthetics)^^82^^.

The decision to maintain CPAP therapy during the pandemic must be accompanied by adherence to the relevant safety rules. Due to the risk of aerosolization, the patient should sleep in a separate room. It is important that they wash their hands before positioning the device at night and after use. The CPAP mask and humidifier require daily cleaning with water and mild soap, and be allowed to air dry. The usual recommendation for the cleaning of headgear and hose cleaning is weekly, but some professionals recommend that this should be done daily during the pandemic. Specific routine cleaning should follow the manufactures’ instructions, and the room must be kept well ventilated. 

At the moment, the initiation of CPAP therapy should be reserved for severe cases only. Patients with a recent diagnosis of mild or moderate OSA have probably already had an apnea for months, or even years. Thus, CPAP can be postponed, and alternative therapies should be used. A temporary suspension of CPAP therapy may be advisable for patients unable to sleep in a separate room. It should be noted that stopping CPAP may be associated with the recurrence of symptoms, such as excessive daytime sleepiness, headaches, and impaired concentration. Kohler et al. (2011)^^83^^ demonstrated that two weeks of CPAP withdrawal was associated with impairment of endothelial function, an increase in urinary catecholamines, blood pressure, and heart rate^^83^^, which may be harmful in respect of cardiovascular risk. Patients stopping CPAP during the pandemic must be cautioned to remain aware of any deterioration in their physical and mental health, and should be re-evaluated regularly by their sleep specialist.

### Insomnia management during COVID-19 pandemic

The possible effects that SARS-CoV-2 might have on the mental health of patients, health professionals, and the general public of SARS-CoV-2 has been a matter of increasing concern. Chronic isolation, fear, economic uncertainties, and stress are among potential risk factors for the development or worsening of insomnia, anxiety and depression. All these variables are associated with higher psychological distress^^84^^, making individuals more prone to insomnia or poor sleep quality^^85^-^87^^.

Most research, so far, has focused on health professionals, with studies reporting increased depression/depressive symptoms, anxiety, psychological distress, poor sleep quality^^88^^, and insomnia symptoms during the COVID-19 outbreak^^89^,^90^^. Studies investigating COVID-19 patients, found a high level of posttraumatic stress symptoms and depressive symptoms, including the worsening of symptoms of preexisting psychiatric disorders^^8^,^16^,^19^^. Studies of the general public, revealed lower psychological well-being, and higher anxiety and depression scores compared to before COVID-19^^8^,^9^,^19^^.

Worries included loneliness, education status, being infected by the virus, and pre-existing mental health illness were major contributing variables to clinical insomnia, which is an important component of mental health interventions during COVID-19 pandemic^^91^,^92^^. Insomnia severity is independently associated with elevated suicidal ideation and should, thus, be carefully monitored. Non-pharmacological approaches, such as cognitive-behavioral therapy for insomnia, are highly effective and recommended as first line treatments^^93^^. There are a number of cognitive-behavioral therapy for insomnia programs available, including those delivered through the internet or telemedicine when social isolation is required. Some simple recommendations related to good sleep hygiene such as maintaining regular sleep-wake schedules, and being active during the daytime (including some physical activity) may help to mitigate the effects of insomnia and/or poor sleep quality. Physical activity may have some effect on wellbeing and the immune system^^94^^. Although there are a number of drugs available for chronic and short-term insomnia that have been approved by the US Food and Drug Administration (FDA) and other regulatory agencies, care is advised when prescribing psychoactive drugs to patients, especially during COVID-19 pandemic^^95^^. The prevalence of insomnia has clearly increased during the COVID-19 pandemic, and further studies are needed to establish what the best care and prevention measures are, particular among medical staff.

### Restless legs syndrome and periodic limb movement disorder during COVID-19

Restless legs syndrome (RLS) and periodic limb movement disorder (PLMD) are classified as sleep-related movement disorders. The prevalence rate ranges from 3.9 to 15% of the general population in recent epidemiological analyses from different countries^^96^^. The pathophysiology of RLS/PLMD is still only partially understood. The most accepted mechanisms include genetic variants, abnormal iron metabolism, dopaminergic dysfunction, and the involvement of the central opioid system^^97^^.

Sleep deprivation, alcohol or tobacco use, reduced motility, medications (dopamine antagonists, antihistamines or serotonergic antidepressants, discontinuation of opioids), metabolic changes^^98^^ and mood changes^^99^^ can result in the onset of RLS, or an increase in the severity of its symptoms^^100^^. The main drugs to treat RLS are gabapentin, enacarbil and pregabalin (α2-δ GABA ligands); and pramipexole; ropinirole and rotigotine (all three dopaminergic agonists), all in low doses^^101^^. However, there are variations in dose or in the effects and, for some severe cases, opioids may be prescribed^^102^^, i.e., the oral or intrathecal administration of morphine for selected patients with chronic pain^^103^^, not responsiveness for the drugs mentioned above.

The interaction between these drugs described above, as well as the drugs used to treat COVID-19 are unknown, mostly due to the lack of a standard protocol to treat patients with RLS or PLMD, whose may have been receiving treatment for COVID-19. Caution is required in the rare cases in which opioids are used, due to respiratory depression risk^^104^^.

## COVID-19 and circadian rhythmicity

### Circadian rhythm misalignment

Circadian rhythms coordinate most biological processes^^84^^, which are regulated by endogenous and peripheral clocks^^85^^. The circadian modulation may occur by different stimuli, such as body temperature, sleep/wake cycles, and hormone secretion^^86^^. Any misalignment in circadian rhythms can, therefore, disrupt a range of biological processes^^84^^. Chronic or recurrent patterns of sleep-wake rhythm disruption are known as circadian rhythm disorders, and are primarily due to alterations in the endogenous circadian timing system, or misalignment between endogenous circadian rhythm and the sleep-wake schedule desired, or required, by an individual’s physical environment or social/work schedules. The symptoms include insomnia, excessive sleepiness or both, leading to clinically significant distress or impairment in mental, physical, social, occupational, educational or other important areas of functioning^^79^^.

Regularity of the sleep wake cycle is important for optimizing health, and the function of the circadian system and biological clock. As mentioned previously, it is not only insufficient sleep, but also irregular sleep-wake schedules that can impair health, the immune system, increase inflammation, and perhaps lead to increased vulnerability to viral infections, such as COVID-19. The host circadian clock has now been recognized as a major modulator of immune/inflammatory responses in general^^19^-^21^^, and in particular following respiratory virus infections *in vivo*. It has been shown that an endogenous circadian clock within lung epithelial cells modulates neutrophil recruitment through the chemokine ligand (CXCL5). The molecular clock controls host cell responses to influenza virus infection via natural killer (NK) cell functions^^105^^.

Extensive literature has shown the important role of homeostatic sleep in innate and adaptive immunity^^62^^. In the context of treatment during SARS-CoV-2 infection, it may require more precise alignment with the patient’s circadian clock status^^105^^. As an example, melatonin is able to align the circadian system, and has been demonstrated to relieve many of the symptoms of other viral infections. It may have some beneficial effect also in patients with COVID-19 infection^^106^,^107^^.

COVID-19 patients and health professionals acting in the front are at risk for developing circadian rhythm problems require marked attention. The significant changes in daily routine, sedentarism, possibility of daytime naps, irregular bedtime, and wake up times, increases the risk of sleep wake disorder. However, exposure to shifts, stress and night or shift-work add significant higher risk to health professionals. A recent study showed that poor-sleep quality in patients with COVID-19 was associated with a slow recovery from lymphopenia, an increased risk of becoming critically and requiring intensive unit care, and a longer hospital stay^^108^^.

Good sleep hygiene may help to avoid disruption to the sleep wake cycle. This includes measures, such as being active during the day, getting sufficient exposure to natural light, socializing (being isolated does not mean necessarily to be alone), avoiding excessive light exposure/electronic devices close to bedtime, going to bed and waking at set times, and maintaining a regular schedule for meals^^109^^. In this context, sleep disturbances may facilitate SARS-CoV-2 infection in shift-workers, due to the fact of shift-work result in sleep restriction (SR), and contribute to the risk of developing sleep disturbances^^110^^, and circadian desynchronization^^111^^. It may result in increased inflammation^^112^^, immune system deregulation^^113^,^114^^, and increased lapses in attention^^115^^, negatively effects metabolic response^^116^^, particularly in relation to the levels and rhythms of stress hormones, such as cortisol^^111^^ and melatonin^^117^^, as well as inflammatory changes^^118^^ include reduced lymphocyte proliferation^^119^^, especially natural NK cells^^120^,^121^^; increased levels of leukocytes^^122^,^123^^; monocytes (IL-6, IL12, IL-17, TNF-α)^^41^,^124^^; and nuclear factor-kappa B (NF-kB)^^125^,^126^^.

**Figure 1:**
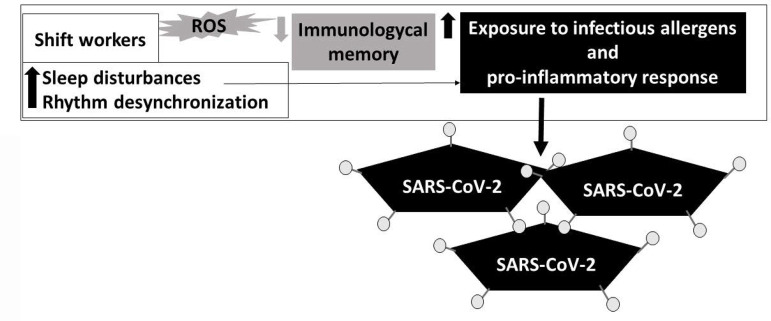
Sleep disturbances may facilitate SARS-CoV-2 infection in shift-workers. Illustrative scheme shows that shift workers present pro-oxidant/anti-oxidant imbalance, inducing oxidative stress and elevating the levels of reactive oxygen species (ROS), as well as a reduced immunological memory, and increased exposition to allergens. They also present a pro-inflammatory state and, mostly, sleep disturbances, which may facilitate SARS-CoV-2 infection.

Shift-work can alter the pro-oxidant/anti-oxidant balance, inducing oxidative stress and elevating the levels of reactive oxygen species (ROS)^^127^^. Is important highlight that the immunological memory may be impaired in shift-workers^^128^^. Furthermore, shift and night shift-workers are often exposed to many inhaled allergens, and their tolerance to chemical, physical, and biological or infectious agents may be impaired because of the time of exposure^^129^^, as at night individuals generally present a pro-inflammatory state and an anti-inflammatory state during the day^^130^^. This may be further compounded by the presence of sleep disturbances. Considering this evidence, we understand that sleep disturbances may facilitate SARS-CoV-2 infection in shift-workers and exacerbate the immune virus response^^131^,^132^^, as is shown in Figure 1.

## Physical exercise and sleep during COVID-19 pandemic

Physical exercise is considered an important non-pharmacological method to treat sleep disorders^^133^,^134^^, anxiety^^135^^, and some chronic diseases^^136^^, with factors such as duration, volume, intensity, and type of exercise influencing its effect^^134^-^136^^. Considering the benefits promoted by physical exercise, the increase in this practice has been encouraged during the COVID-19 self-quarantine^^137^^. However, it is necessary to be careful with this recommendation because the practice of high intensity physical exercise may induce immunodepression, which can persist for up to 72 hours^^138^,^139^^.

Before starting physical exercise practice, it is recommended to evaluate the parameters of its volume and intensity, as well as the duration of the training program to obtain the maximum benefit. The type of exercise also needs to considered, as does its suitability for particular groups.

A study conducted with sedentary individuals, concluded that a single session of resistance exercise - with a 50% load of 1 maximum repetition - was not enough to improve sleep parameters assessed using actigraphy^^140^^. Another study compared the acute effects of a session of different types of physical exercise (moderate-intensity aerobic, intense interval aerobic, and moderate-intensity resistance exercise) on sleep patterns^^141^^. The results showed that a single session of physical exercise, regardless of type or intensity, did not change the sleep patterns of sedentary individuals. However, aerobic training of moderate-intensity, performed for 60 minutes, three times a week for 6 months, improved the sleep profiles of older adults measured by polysomnography^^142^^. Another study reported similar results^^143^^. Thus, in individuals with good sleep quality, the benefit of the training is only achieved with regular practice^^142^^.

Other studies have described the beneficial potential of physical exercise to improve sleep parameters in individuals with sleep disorders^^144^,^145^^. Aerobic exercise has been reported to be beneficial for patients diagnosed with a periodic leg movement disorder^^146^^. For individuals diagnosed with periodic leg movement^^147^^, both acute physical exercise (cycle ergometer - 50min, intensity close to the anaerobic threshold), and chronic physical exercise can improve sleep efficiency, REM sleep, and reduce awakenings after sleep onset assessed by polysomnography.

An important review concluded that physical exercise is an effective strategy to reduce sleep complaints and chronic insomnia^^145^^. A study was conducted to compare the acute effect of three modalities of physical exercise (moderate-intensity aerobic exercise x high-intensity aerobic exercise x moderate-intensity resistance exercise) on the pattern of sleep and anxiety in patients with chronic insomnia^^148^^. Moderate-intensity aerobic exercise was the only one reported to be capable of reducing anxiety and sleep latency, and increasing efficiency and total sleep time assessed by polysomnography^^148^^. In respect of long-term training, a study found that six months of moderately intensive aerobic exercise significantly improved sleep, mood, and quality of life in individuals with chronic insomnia^^149^^. Similar benefits were seen in symptoms of depression^^150^^. Aerobic exercise for two months showed positive results, helping to treat patients with OSA syndrome, reducing drowsiness, and improving quality of life and mood^^151^^. Another study showed that in individuals with insomnia, four months of resistance training or stretching (60 minutes, three times a week) improved sleep parameters evaluated by actigraphy and the Pittsburgh sleep quality index^^152^^.

The benefits of physical exercise as a non-pharmacological treatment method for sleep disorders^^133^,^134^^, anxiety^^135^^, and other chronic diseases^^136^^ are well supported by the literature. The practice of physical exercise during the COVID-19 pandemic should be encouraged^^137^^. However, it should be emphasized that physical exercise can be beneficial or harmful, depending on its intensity^^153^^. High-intensity exercise might be avoided in order not to compromise the immune system^^139^^. The practice of physical exercise at moderate intensity, performed for 60 minutes, whether aerobic, resistance or stretching^^148^-^150^,^152^^, has already been shown to be beneficial for improving sleep, quality of life, mood^^149^^, anxiety^^148^^ and the symptoms of depression^^150^^, and, is thus, recommended in the current situation.

## Future perspectives

The future after the COVID-19 pandemic is uncertain, and there are many doubts regarding pathophysiological, epidemiological, and treatment issues - particularly in respect of the different patterns of the disease, the policies of countries to deal with it, and the possibility of new waves of infections. Although epidemiologists are devoting great efforts to develop effective predictive models, it is difficult to fully understand the path of the disease in the near future^^154^^. The question of whether sleep-related changes will be limited to the period of the pandemic and social isolation, or if they will persist when social routines return to normal, remains open. Although the future of sleep medicine in these pandemic times is unclear, some tendencies can be observed, as outlined below:

*Telemedicine*: previous studies have demonstrated that telehealth can be effective in public health emergencies and major disasters^^155^^, and it has been widely adopted during the COVID-19 pandemic^^156^^. Remote consultations and appointments have become more frequent in all areas, including in sleep medicine. Even before COVID-19, sleep-related remote appointments were reasonably common among psychologists, mainly in respect of the application of cognitive-behavioral therapy, and in the monitoring of patients using positive pressure devices^^157^^. However, due to the demands of social isolation, this has expanded to other fields. This experience has been positive in most cases. In person, appointments are more effective in some cases, especially in respect of clinical examination; however, telemedicine is valuable tool for follow up appointments.

Legal and regulatory aspects might be a barrier to the implementation of telemedicine in some countries. The field of sleep medicine is going through important changes during the pandemic. The rapid transition to delivering training programs online and patient care through remote consultation because of the risks of contamination is not easy but is unavoidable. The use of telecommunication technologies, electronic devices, and services to provide care at-a-distance had already become more established in sleep medicine than in many other specialties^^157^^. Home sleep tests and follow-up strategies were already common in sleep medicine. However, the pandemic, and its consequences, forced the acceleration of the employment of these techniques in many countries. 

Teleconference facilities are being used to discuss clinical cases and new sleep studies. The American Academy of Sleep Medicine is offering updates on the latest findings related to COVID-19 and sleep through clinical conversations with experts. These difficult times can be turned into an opportunity to increase the integration of the multidisciplinary areas of sleep medicine. Sleep medicine research has been impacted globally, and there is a need to establish what research priorities should be to continue to advance knowledge in this field. International collaboration and the use of big data will be essential to target the scientific questions that will emerge from the outbreak^^158^^. 

*Home monitoring*: considering the restrictions on in-lab polysomnography, home monitoring for OSA (from type II to IV) have been stimulated. Although we encourage the use of home monitoring for OSA, in-lab monitoring will still play an important role and cannot be completely replaced by home monitoring.

*Insomnia:* several circumstances related to the COVID-19 pandemic are potential precipitators and perpetuators of insomnia (including anxiety, home confinement, and uncertainty). In these cases, it is possible that many individuals will present persistent insomnia, even when back to their normal routine, considering the possible damage related to mental health already mentioned above^^19^,^91^^. The infection can play a role in the appearance of sleep disorders in survivors after SARS-CoV-2^^159^^. Whether this will lead to significant impact on the prevalence of insomnia is an open question. 

*OSA:* it has been suggested that OSA is a possible risk factor for negative outcomes of COVID-19^^67^^, rather than a consequence of it. We do not expect the prevalence of OSA to increase due to COVID-19. However, some reports have addressed the increased risks of weight gain during home confinement and self-quarantine^^7^,^160^^, which might lead into an increased likelihood of developing OSA.

*Circadian rhythm disorders:* home confinement and the practice of working from home might increase the likelihood of rhythm disorders, especially delayed sleep-wake phase disorders. These patients might have difficulty in readapting to regular work schedules and should be advised to keep regular routines while in home confinement. An adaption period, in which the patient can progressively readapt to normal schedule is recommended.

## CONCLUSION

The measures adopted in response to the COVID-19 pandemic, such as social distancing, home confinement, changes in work patterns, and home schooling; combined with job uncertainty and health worries, has resulted in higher levels of anxiety, depression, and stress, increasing the mental health burden and affecting sleep patterns, disrupted daily life and may have a significant effect on sleep health and the development of sleep disturbances. At the same time, most sleep centers had to close and stop on-site activities and consultations.

Although the full data is not yet available for SARS-COV-2, it is expected to be a significant increase in insomnia and other sleep disorders. Many patients had to suspend treatments for sleep disorders during this period. COVID-19 pandemic has been associated with significant circadian rhythm changes. While it gave some individuals the opportunity to follow their natural circadian rhythms, resulting in improved sleep, others presented circadian misalignment and sleep disturbances, especially night shift-workers. In this sense, a disruption of the circadian rhythm has the potential to decrease the response of the immune system and increase the risk of infection, highlighting the importance to maintain healthy sleep habits, as well as regular wake and sleep times to preserve normal circadian biology. 

COVID-19 has affected sleep medicine, which may return to its previous regular processes when the pandemic is over. Even if this is the case, the pandemic period should be seen as a time for learning and reflection, reinforcing how important regular and healthy sleep habits are, as well as how susceptible sleep is to environmental, social, and behavioral circumstances.
